# The Lipid-Lowering Efficacy of a Nutraceutical Combination Including Leucoselect Phytosome, Red Yeast Rice, Policosanol and Folic Acid in Dyslipidaemia Patients: Real-World Insights

**DOI:** 10.3390/ph17040447

**Published:** 2024-03-30

**Authors:** Vincenzo Russo, Nicola Napolitano, Antonia Ascrizzi, Silvia Leonardi, Filomena Pisacane, Pierpaolo Di Micco, Egidio Imbalzano, Ferdinando Carlo Sasso, Antonello D’Andrea, Alfredo Caturano, Alfredo Mauriello

**Affiliations:** 1 Cardiology Unit, Department of Medical Translational Sciences, University of Campania “Luigi Vanvitelli”, Monaldi Hospital, 80131 Naples, Italy; napnic@tin.it (N.N.); antonia.ascrizzi@gmail.com (A.A.); alfredo.mauriello93@libero.it (A.M.); 2 Clinical Biochemistry Unit, Monaldi Hospital, 80131 Naples, Italy; 3 Department of Medicine, Presidio Ospedaliero Santa Maria delle Grazie, 80078 Pozzuoli, Italy; pdimicco@libero.it; 4 Department of Clinical and Experimental Medicine, University of Messina, 98122 Messina, Italy; 5 Department of Advanced Medical and Surgical Sciences, University of Campania “Luigi Vanvitelli”, 80138 Naples, Italyalfredo.caturano@unicampania.it (A.C.); 6 Department of Cardiology, Umberto I Hospital, 84014 Nocera Inferiore, Italy

**Keywords:** cardiovascular risk, cholesterol, nutraceuticals, adherence, leucoselect phytosome, red yeast rice, policosanol, berberine, folic acid, safety profile

## Abstract

Background: Cardiovascular disease is a global health concern and reducing plasma LDL-C levels is a major goal in cardiovascular prevention. Our study aimed to evaluate the effectiveness of a nutraceutical formulation including leucoselect^®^ phytosome^®^, red yeast rice, policosanol and folic acid on LDL-c levels in patients at low cardiovascular risk with dyslipidemia. Materials and Methods: We prospectively enrolled all consecutive patients with dyslipidemia at low cardiovascular risk who were unresponsive to diet and physical activity. Clinical assessments and laboratory analyses, encompassing lipid profile, hepatic function, and CPK levels, were performed at baseline prior to initiating treatment and repeated at the 12-week mark following administration of the study nutraceutical. Results: Sixty (60) consecutive patients (mean age 48.02 ± 10.1 years; 60% male) were included. At the 12-week follow-up, a statistically significant reduction in Total Cholesterol (13.1%) and LDL-c serum level (20.4%) was observed. Hepatic and muscular function remain stable over the time. The adherence to therapy was 99% and the persistence was maximum. Conclusions: The nutraceutical formulation including leucoselect^®^ phytosome^®^ red yeast rice, policosanol and folic acid significantly reduced the LDL-c plasma levels, consistent with previous research showing that the bioactive component in red yeast rice—lovastatin—is effective in addressing problems with lipid metabolism. Importantly, it was safe and well-tolerated among patients with dyslipidemia in a real-world setting.

## 1. Introduction

Cardiovascular diseases (CVDs) include several heterogeneous conditions mainly due to the atherosclerosis and are considered the leading cause of premature mortality and morbidity worldwide to date [1,2,3]. Atherosclerosis is a complex immuno-inflammatory process in which the low-density lipoprotein cholesterol (LDL-c) plays a role of pivotal importance in the genesis, progression and rupture of the lipid plaque; this latter condition may lead to thrombus formation, myocardial infarction and stroke [4,5,6,7]. The role of LDL-C as a causal factor in atherosclerosis is widely acknowledged. Specifically, the impact of LDL-C on the risk of atherosclerotic cardiovascular disease (ASCVD) is influenced by both the absolute level and the cumulative duration of exposure to LDL-C [8]. Moreover, several studies showed a direct relationship between plasma levels of LDL-c and the occurrence of cardiovascular acute events [9,10,11,12]. Reducing LDL cholesterol (LDL-C) levels in the blood proportionally decreases the risk of ASCVD, correlating with the absolute reduction achieved in LDL-C levels [8,10]; consequently, a main target of the cardiovascular prevention is to reduce the plasma level of LDL-c according to the individual risk.

The current European Society of Cardiology (ESC) guidelines recommend assessing the total cardiovascular risk using a calibrated country-specific version of the SCORE (Systematic Coronary Risk Estimation) system [13]. Patients are considered at low risk when the calculated SCORE is < 1% for 10-year risk of fatal CVD; among this subgroup, a serum level of LDL-c < 116 mg/dL may be considered to reduce the major vascular events; consequently, it is highly recommended to adopt healthy lifestyle measures, such as maintaining healthy eating habits, engaging in regular exercise, and abstaining from smoking [14].

In line with the ESC guidelines, which emphasize the importance of comprehensive cardiovascular risk assessment and lifestyle modifications, recent attention has turned to the potential role of nutraceutical interventions in managing dyslipidemias. Several studies have explored the efficacy of various nutraceutical combinations in addressing lipid abnormalities [15]. Recently, a novel nutraceutical combination including leucoselect^®^ phytosome^®^ (an association between grape seed standardized extract and lecithin), red yeast rice, policosanol and folic acid (Ostacol Plus, Agaton) has been formulated for the first-line treatment of dyslipidemia in patients at low cardiovascular risk not responsive to diet and physical activity. The aim of our study was to evaluate the effect of this nutraceutical combination on LDL-c serum levels among patients with dyslipidaemia at low cardiovascular risk in a real-world setting.

## 2. Results

Sixty (60) consecutive patients (mean age 48.02 ± 10.1 years; 60% male) were included in the present study. The mean body mass index (BMI) was 25.7 ± 3.1 Kg/m^2^; 16 patients (26%) were smokers (Table 1). All patients showed a basal serum level of LDL-c higher than 116 mg/dL, despite at least 6 months of hypercholesteraemic diet and physical exercise and were treated with Ostacol Plus once daily. The nutraceutical formulation was continued for at least 12 weeks. The nutraceutical intervention exhibited exceptional tolerability, as evidenced by the absence of any adverse events among the study participants. There were no instances of temporary or definitive interruptions reported throughout the duration of the study. Furthermore, the adherence rate to the therapy regimen reached 99%, indicating strong compliance among the participants.

**Figure 1:**
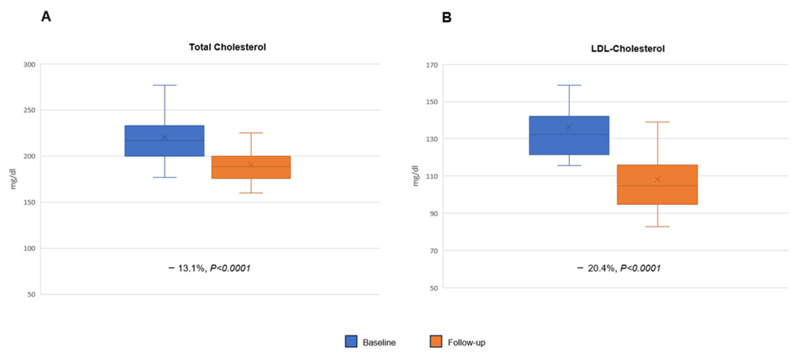
Scatter plots of baseline (blue) and follow-up (orange) plasma levels of total cholesterol (TG) (panel **A**) and LDL cholesterol (LDL-C) (panel **B**).

At 12 weeks follow-up, a statistically significant reduction in TC (219 ± 24.6 vs. 190.3 ± 19.9 mg/dL; −13.1%; *p* < 0.0001) and LDL-c serum level (137.1 ± 19.3 vs. 109.1 ± 18.4 mg/dL; −20.4%; *p* < 0.0001) was shown (Figure 1A,B). No significant changes in TG (133 ± 73.8 vs. 129.3 ± 71.3 mg/dL; *p* = 0.7690) and HDL-c (55.1 ± 15.4 vs. 55.3 ± 13.9 mg/dL; *p* = 0.9378) plasma levels were observed. The AST (23.5 ± 10.6 vs. 24.8 ± 11.1 U/L; +1.34%; *p* = 0.5002), ALT (24.6 ± 10.1 vs. 25.9 ± 10.2 U/L; *p* = 0.5009) and CPK (85.7 ± 37.4 vs. 89.4 ± 40.6 U/L; +4.3%; *p* = 0.6086) serum levels remained stable.

At the end of follow-up period, 45 (75%) patients achieved the LDL-C therapeutic range; the remain 15 patients who did not achieve the therapeutical goals were switched to statin therapy.

**Figure 2:**
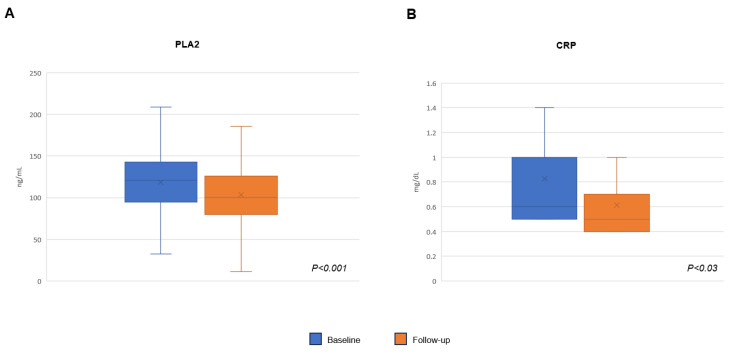
Scatter plots of baseline (blue) and follow-up (orange) plasma levels of phospholipase A2 (PLA2) (panel **A**) and C-reactive protein (CRP) (panel **B**).

A significant reduction in phospholipase A2 (PLA2) was shown (118.48 ± 42.1 vs. 103.5 ± 39.42 ng/mL; *p* = 0.001). Fifteen (15) patients showed high CRP serum levels at baseline with a significant reduction at 12-week follow-up (0.82 ± 0.45 vs. 0.60 ± 0.28 mg/dL; *p* = 0.03); among them, seven (46.7%) showed the normalization of the absolute PCR value (<0.4 mg/dL) (Figure 2A,B).

## 3. Discussion

The main findings of the present study are as follows: the use of a nutraceutical formulation including leucoselect^®^ phytosome^®^, red yeast rice, policosanol and folic acid determines a significant reduction of 20% in the LDL-C serum level among patients at low cardiovascular risk with dyslipidaemia not responsive to diet and physical exercise; 75% of treated patients achieved the LDL-C therapeutical level within 12 weeks of treatments. The use of this novel nutraceutical combination is safe and well-tolerated in a real-world setting and it is characterized by maximum persistence and high adherence. A pleiotropic effect on inflammatory biomarkers has been shown.

### 3.1. Nutritional Intervention

The Mediterranean diet, known for its emphasis on plant-based foods like fruits, vegetables, legumes, nuts, and whole grains, as well as the inclusion of healthy fats from sources such as olive oil and fish, is widely recognized for its ability to improve CVD risk factors. Additionally, it has beneficial effects on disease endpoints [16], because it significantly reduces the TC by 11%, LDL-C by 13%, and triglycerides by 7% [17]. Foods rich in unsaturated and low in saturated and trans fatty acids, such as rapeseed or canola oil, supplemented with plant sterols/stanols, and high in soluble fiber from sources like oats, barley, and psyllium, have been shown to induce at least moderate reductions in LDL cholesterol levels. Additionally, consumption of soy protein, tomatoes, flaxseeds, and almonds has been associated with small reductions in LDL cholesterol. Avocados and turmeric have demonstrated moderate to large reductions in LDL cholesterol levels with moderate evidence. Pulses, hazelnuts, walnuts, high-fiber/whole grain foods, and green tea consumption have been linked to small to moderate reductions in LDL cholesterol levels [18]. Combining nutraceuticals with different lipid-lowering activities, along with a healthy lifestyle, presents a potential alternative to medication for individuals in primary prevention of CVD who have mildly elevated LDL-C levels [19]. This approach is especially beneficial for patients who have not reached their LDL-C target and for some statin-intolerant patients [19].

### 3.2. Lipid Lowering Nutraceuticals

Nutraceuticals with lipid-lowering properties play an important role in CVD prevention among patients at low cardiovascular risk with dyslipidaemia not responsive to diet and physical exercise [20]. Among them, the most studied are monacolin of fermented red rice, polycosanol and berberine [21].

**Figure 3:**
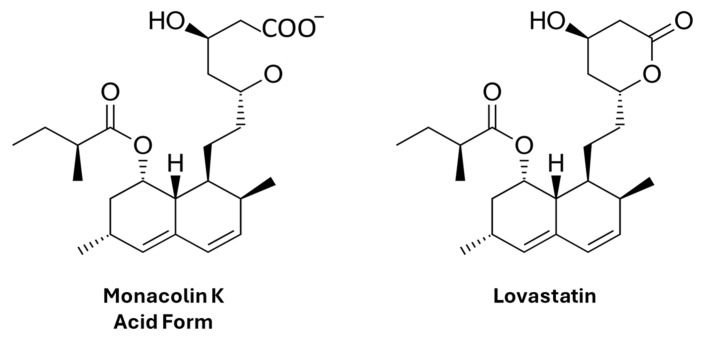
Structure of monacolin k and lovastatin.

Red yeast rice (RYR) is a nutraceutical made from fermenting a specific yeast in white rice, resulting in a red color [22]; the fermentation process enriches the rice with compounds that have lipid-lowering properties, such as monacolins [23]. Among them, the monacolin K (MonK) has a similar biochemical structure of the lovastatin (Figure 3) and works by inhibiting HMG-CoA reductase, an enzyme involved in cholesterol production [24,25]. Lovastatin is a statin derived by the fungus *Aspergillus terreus* (ATCC 20542) [26]. Nevertheless, despite sharing a similar structure, their pharmacological profiles exhibit differences. Lovastatin is considered a pro-drug, existing as an inactive gamma-lactone in its native state. However, within the body, it undergoes hydrolysis to the active beta-hydroxy acid open-ring form, which is the most bioavailable and pharmacologically active. In RYR, the ratio of monacolin K lactone to acid varies significantly, ranging from 5% to 100% of the total monacolin K content. This variation strongly influences the molecule’s bioavailability. The process of lactone ring opening can occur through metabolism in alkaline conditions or enzymatically by the cytochrome P450 3A family in the small intestine and liver [27]. Additionally, recent evidence suggests that gut microbiota do not convert monacolin K into the beta-hydroxy acid form but instead catabolize it. This indicates that gut microbiota may impede the lipid-lowering effects of both lovastatin and monacolin K by degrading their active metabolite [28].

A meta-analysis of 20 randomized controlled trials found that supplementation with RYR for 2–24 months led to an average reduction of 1.02 mmol/L in LDL cholesterol compared to placebo [29]; moreover, RYR increased HDL-C and reduced triglycerides when compared to placebo [29]. Several studies have shown that RYR can also improve inflammatory biomarkers, endothelial function, and pulse wave velocity [29].

Polycosanols are a mixture of saturated aliphatic primary alcohols derived from various sources such as sugar cane wax, beeswax, rice bran, and other plants [30]. The main components of this mixture are octacosanol, triacontanol, hexacosanol, and other heptacontanols [21]. Its supplementation inhibits cholesterol biosynthesis by activating 5′ adenosine monophosphate-activated protein kinase (AMPK) and deactivating 3-hydroxy-3-methylglutartl coenzyme A (HMG-CoA) reductase. Moreover, it facilitates the excretion of cholesterol and bile acids while augmenting the uptake of LDL cholesterol by LDL receptors in the bloodstream, especially in the context of hypercholesterolemia [31]. However, it is important to recognize that the effectiveness of polycosanols can vary among individuals due to the different polyalcohols present in the mixture. Although clinical studies have shown a 20% decrease in LDL-C, more recent studies have raised questions about their ability to lower lipids in different populations [32]. Tolerability appears to be good in the short and medium term [32]. The oral bioavailability of Policosanol, its sole route of administration, is typically below 10%. However, research suggests that its absorption can be enhanced through methods such as nanoemulsification [33] or esterification with oleic acid [34].

Berberine is a natural alkaloid that is found in various medicinal plants. Its mechanism of action is not fully understood, but it primarily targets the liver and affects various metabolic structures [21]. One potential mechanism of action is the inhibition of proprotein convertase subtilisin/kexin type 9 (PCSK9). This mechanism extends the half-life of hepatic LDL-receptors, responsible for capturing LDL-cholesterol from the bloodstream. Another potential pathway involves the activation of the AMPK, which regulates energy balance and lipid synthesis [35,36]. Another proposed mechanism is the inhibition of aldo-keto reductase family 1 member C3 (AKR1C3), which modulates steroid hormone action by converting estrone to 17β-estradiol or androstenedione to testosterone [37]. Berberine does not exhibit dose-dependent effects, as a daily intake of 500 mg can already reduce LDL-cholesterol by approximately 20% and triglycerides by 25% [38]. Berberine has demonstrated positive effects on insulin resistance in individuals with various conditions, including hypercholesterolemia, mixed dyslipidemia, type 2 diabetes, and hepatopathies [21]. Overall, it exhibits potential as a natural alternative for managing cholesterol and metabolic disorders [21]. Even though berberine exhibits low systemic bioavailability following oral administration due to its poor absorption in the gut and rapid metabolism within the body [39], in vivo studies have demonstrated significant biological activities of berberine, with its major metabolites including berberrubine, thalifendine, demethyleneberberine, and jatrorrhizine being present at relatively high concentrations [40,41].

Polyphenols are a group of functional food ingredients derived from plants. These nutraceuticals possess distinct molecular structures and exhibit a diverse range of biological activities, encompassing antioxidant, anti-inflammatory, and anticancer properties, among others [42]. Red wine contains phenolic compounds that increase antioxidant activity in the blood [43], preventing the oxidation of LDL cholesterol and delaying the formation of atheroma [44]. Research suggests that red wine can protect LDL from oxidation [45] due to the antioxidant capacity of these phenolic compounds [46], including grape procyanidins that act as free radical scavengers and work together with other antioxidants like vitamin E [47]. Polyphenols can be extracted and purified from grape seeds to create a standardized pharmaceutical preparation. In a study, a dosage of 300 mg of polyphenols from grape seed extract called leucoselect^®^ phytosome^®^ was shown to increase total antioxidant activity in the blood without impact on levels of serum vitamin E and C [48]. Moreover, phytosomes, among vesicular drug carriers, facilitate the formation of a complex between phytochemicals and phospholipids. This interaction leads to enhanced absorption and bioavailability of bioactive molecules, accompanied by improved overall compound stability [49,50].

### 3.3. Clinical Studies

Numerous clinical investigations [51] have explored the lipid-lowering efficacy of nutraceuticals in individuals with mild to moderate dyslipidemia, revealing a spectrum of LDL-C reduction rates ranging from 19.8% to 25.7%, depending on the specific clinical context [51,52]. Notably, a combination formulation comprising red yeast rice, policosanol, berberine, folic acid, astaxanthin, and coenzyme Q10 (marketed as Armolipid Plus^®^, AP) has garnered significant attention across various studies [53,54,55,56,57,58]. These studies collectively demonstrated reductions in total cholesterol (TC) and LDL-C levels spanning from 10% to 20% and 15% to 31%, respectively. Moreover, in patients intolerant to statins or those unable to achieve target lipid levels with ezetimibe, AP supplementation exhibited promising results, yielding an additional 10% improvement in TC and LDL-C levels [59]. Importantly, adverse events associated with AP therapy were infrequent, with only 2.2% of patients reporting adverse effects, primarily consisting of constipation (0.5%), elevated creatine phosphokinase (CPK) levels > 5 times the upper limit of normal (ULN), myalgia, and dyspepsia [59]. These findings underscore the favorable safety profile and potential clinical utility of AP as an adjunctive therapy in the management of dyslipidemia, particularly in statin-intolerant individuals or those with suboptimal lipid control despite standard therapy.

Few studies [60,61,62,63,64] evaluated the effect of nutraceuticals on inflammatory biomarkers with contrasting findings. The regular intake of intake of PS-enriched foods did not significantly change CRP, whilst LDL-C concentrations were significantly reduced [65]. In contrast, a nutraceutical combination including phytosterols, red yeast and hydroxytyrosol had beneficial effect on CRP [65]. Lovastatin, like other statins, has been shown to improve CRP plasma levels [66]. However, whether this effect extends to compounds derived from RYR remains a matter of debate [66]. The inflammation plays a key role in the genesis of CVD [7,67,68,69,70,71,72,73,74,75] and cardiovascular drugs with pleiotropic anti-inflammatory effects showed a beneficial impact on cardiovascular mortality [7,67,68,69,70,71,72,73,74,75]. The results of our study suggest that nutraceutical combination of leucoselect^®^ phytosome^®^, red yeast rice, policosanol and folic acid is effective in term of LDL-C serum level reduction and shows an anti-inflammatory effect, reducing both the CPR and PLA2. The explanation of this pleiotropic activity is based on leucoselect^®^ phytosome^®^ [48].

The antioxidant power of grape seed has been tested in human studies [50,75,76,77]. Administering daily doses of the active ingredient (300 mg) twice a day to 20 young volunteers resulted in an increase in the total antioxidant capacity (TAC) among the treated individuals [48]. Additionally, incorporating grape seed extract into a meal regimen has been shown to mitigate postprandial oxidative stress by reducing oxidants and boosting antioxidant levels in plasma [78].

Encouraging but inconclusive findings arose from a randomized, double-blind, crossover study involving 24 healthy heavy smokers. In this study, participants were administered 75 mg of the active ingredient twice a day for a duration of 4 weeks. Following this regimen, individuals taking leucoselect^®^ phytosome^®^ exhibited a significant decrease in thiobarbituric acid-reactive substances (TBARS), indicating reduced oxidative stress [75]. Leucoselect^®^ phytosome^®^ is a formulation combining standardized grape seed extract and soy lecithin, featuring a high concentration of oligomeric proanthocyanidins (OPCs) obtained from the seeds of Vitis vinifera L [79]. The chemical composition is well-defined: 80% (−)-epicatechin gallate, dimers, trimers, tetramers and their gallates, 15% (+)-catechin, (−)-epicatechin, 5% pentamers, hexamers, heptamers and their gallates [62].

In order to enhance their bioavailability, leucoselect^®^ has been reformulated with soy phospholipids at a ratio of approximately 1:2.6 (*w*/*w*), resulting in the creation of leucoselect^®^ phytosome^®^ [80]. The antioxidant molecules act as powerful scavengers of free radicals and contrast the oxidation of low-density lipoprotein cholesterol (LDL-C), helping to preserve the vessels free from plaques [81]. Its cardiovascular protecting activity is supported by four clinical trials, and by extensive pharmacological data [48,75,76,77].

The current investigation presents several limitations that warrant considerations. The observational nature of the study lead to potential biases and confounding factors may have influenced the observed outcomes; the lack of control group and the small number of included patients limited our ability to evaluate the magnitude of the LDL-c reduction among the study population. The short-term follow-up, limited to 12 weeks, did not allow us to evaluate the maintenance in LDL-c lowering over the time. However, this is the first real-world clinical experience on the use of a nutraceutical combination including leucoselect^®^ phytosome^®^, red yeast rice, policosanol and folic acid which evaluated both the lipid lowering effect and the antinflammatory proprieties. A randomized controlled trial is needed to validate our preliminary findings and to explore the potential interplay among the effects of each nutraceutical included in the formulation used in our study.

Finally, despite the potential insights offered by quantifying monacolin K in the nutraceutical using liquid chromatography mass spectrometry and establishing a calibration curve, this information was neither provided by the manufacturer nor independently conducted in our study.

Looking ahead, further research in the field of nutraceutical interventions holds promise for advancing our understanding and management of dyslipidemia and cardiovascular risk. Future studies could delve into elucidating the mechanistic underpinnings of the observed lipid-lowering and pleiotropic effects of nutraceutical formulations, shedding light on their potential synergistic interactions and long-term impacts on cardiovascular outcomes. Additionally, exploring the efficacy of combined nutraceutical and pharmacological approaches may offer novel strategies for optimizing lipid management in diverse patient populations, including those with refractory dyslipidemia or heightened cardiovascular risk. Furthermore, investigations into personalized approaches, considering individual genetic predispositions and metabolic profiles, could pave the way for tailored nutraceutical interventions tailored to specific patient needs. Overall, continued research efforts in this area hold the potential to reshape clinical practice guidelines and enhance patient-centered care in the realm of cardiovascular health.

## 4. Materials and Methods

**Figure 4:**
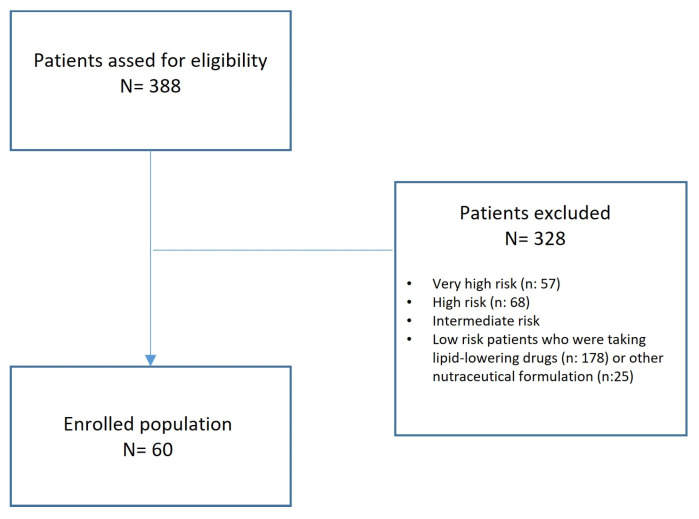
Study flow-chart.

This is a prospective single-arm observational study that enrolled all consecutive patients with an established diagnosis of dyslipidaemia, not responsive to diet and physical activities, and considered at low cardiovascular risk according to ESC guidelines [82]. The inclusion period was from January 2023 to March 2023. Patients who were taking lipid-lowering drugs or other nutraceuticals were excluded (Figure 4).

All patients were treated a novel nutraceutical formulation (Ostacol Plus, Agaton srl, Avellino, Italy) once daily. This formulation included: leucoselect^®^ phytosome^®^ 300 mg, red yeast rice extract: 99 mg, monacolin K 2.97 mg (approximately), polycosanols 10 mg, octacosanol 9 mg (approximately), folic acid 200 mcg [83].

Clinical evaluation and blood laboratory tests, collected after an 8 h overnight fast, were conducted at baseline, before starting treatment, and 12 weeks after study drug administration. The laboratory assessment included including lipid profile (Total Cholesterol (TC), LDL-C, high-density lipoprotein cholesterol (HDL-C), and tryglicerides (TG)), hepatic profile (Alanine Aminotransferase (ALT) and Aspartate Aminotransferase (AST) and muscular status (Creatine Phosphokinase (CPK)), C-reactive protein (CPR) and phospholipase A2 (PLA2).

The primary outcome was to assess the reduction of the serum levels of LDL-c at the end of the observational period. The secondary outcomes included the effect of this novel nutraceutical combination on TC, HDL, ALT/AST, CPK, CRP and PLA2. Furthermore, the study aimed to evaluate the persistence and adherence to this nutraceutical formulation among the study population.

Patients’ adherence to treatment was measured through the medication possession ratio (MPR), dividing the total number of treatment days by the specific time of monitoring. Persistence to therapy was considered the non-temporary or definitive interruption due to side effects. Written informed consent for data storage and analysis was obtained from all patients. The study was conducted in compliance with the Declaration of Helsinki and its later amendments and received approval from the local ethics committee (Ethical Committee of University of Campania “Luigi Vanvitelli”—Monaldi Hospital; ID 23263/2022; approval date: 15 December 2022).

### Statistical Analysis

The data distribution was examined using both the Kolmogorov–Smirnov and Shapiro–Wilk tests. Continuous variables were presented either as mean ± SD or as median with interquartile ranges, depending on the gaussian distribution. The categorical variables were expressed as percentages. The paired Student’s *t*-test/Mann-Whitney test or the chi-square test/Fisher’s exact test were used for continuous and categorical variables, respectively. To assess the differences between mean data over time, the repeated-measures analysis of variance with Bonferroni’s correction was performed. A two-sided *p* < 0.05 was considered significant for all tests. The visual representation of differences in LDL, HDL, PLA2, and CRP levels was achieved using the scatter plots. Data analysis was performed using SPSS 11.0 software for Windows (SPSS Inc., Chicago, IL, USA).

## 5. Conclusions

A nutraceutical formulation including leucoselect^®^ phytosome^®^, red yeast rice, policosanol and folic acid determines a significant reduction of 20% in LDL-C serum level in patients with dyslipidaemia at low cardiovascular risk not responsive to diet and physical exercise. A pleiotropic effect on the reduction of serum inflammatory biomarkers was shown. In a real-world setting, the use of a nutraceutical formulation including leucoselect^®^ phytosome^®^, red yeast rice, policosanol and folic acid is characterized by commendable safety profile and excellent tolerability, coupled with maximum persistence and high adherence. However, it is essential to recognize that regular monitoring and reassessment of cardiovascular risk factors remain crucial in guiding ongoing management decisions. Future studies should further explore the long-term efficacy and safety of this nutraceutical formulation and its potential role in comprehensive cardiovascular risk reduction strategies.

## Figures and Tables

**Table 1:** Baseline characteristics of study population.

Characteristics	*n* = 60
Age—yr	48.02 ± 10
BMI—Kg/m^2^	25.7 ± 3.1
Heart rate—beats/min	77 ± 18
Systolic blood pressure—mmHg	116 ± 15
Diastolic blood pressure—mmHg	67 ± 11
Total Cholesterol, mg/dL	219 ± 24.6
LDL-C, mg/dL	137.1 ± 19.3
PLA2, ng/mL	118.48 ± 42.1
CRP, mg/dL	0.82 ± 0.45

## Data Availability

The data that support the findings of this study are available on reasonable request from the corresponding author.
